# Impact of Various Treatment Modalities on Long-Term Quality of Life in Cervical Cancer Survivors

**DOI:** 10.7759/cureus.68642

**Published:** 2024-09-04

**Authors:** Pavel Sorokin, Svetlana Kulikova, Andrei Nikiforchin, Elena Ulrikh

**Affiliations:** 1 Gynecologic Oncology, Moscow City Oncology Hospital No. 62, Istra, RUS; 2 Gynecologic Oncology, Almazov National Medical Research Center, Saint Petersburg, RUS

**Keywords:** patient response, chemotherapy (ct), adverse event, complication of treatment, radiotherapy (rt), eortc qlq-c30, qol response scale, quality-of-life, quality of life (qol)

## Abstract

Introduction

Given treatment advancements and the long life expectancy of mostly young patients with cervical cancer, their post-treatment quality of life (QoL) is essential to consider. This study aimed to evaluate the long-term QoL in cervical cancer survivors treated with various approaches.

Methods

We conducted a cross-sectional survey-based study using the European Organization for Research and Treatment of Cancer Quality of Life Questionnaire Core 30 (EORTC-QLQ-C30) and the European Organization for Research and Treatment of Cancer Quality of Life Questionnaire Cervical Cancer Module 24 (EORTC-QLQ-CX24) questionnaires and involved members of the online cervical cancer patient support group (01/2024-02/2024). Eligible participants were ≥18 years old, diagnosed with stage IA2-IIB cervical cancer, and had completed their treatment. Respondents were stratified into four management groups: neoadjuvant chemotherapy + surgery +/- radiation therapy (RT), surgery + RT, RT alone, and surgery alone.

Results

Overall, 173 patients participated: 20 (11.6%) received neoadjuvant chemotherapy + surgery +/- RT, 50 (28.9%) had surgery + RT, 69 (39.9%) had RT alone, and 34 (19.7%) had surgery alone. Patients after surgery alone had significantly better global QoL (p<0.001). Their physical (p<0.001), role (p=0.037), emotional (p=0.024), and social (p=0.006) functioning were also substantially better. This group also reported the lowest severity of fatigue (p=0.001), nausea and vomiting (p<0.001), and diarrhea (p<0.001). Sexual functioning was better in the surgery-alone group in almost all aspects. There were no major differences in QoL among the groups, receiving RT alone or combined with other treatments.

Conclusions

Cervical cancer survivors who underwent surgery alone reported the highest QoL and lower symptom intensity compared to those treated with RT or treatment combinations. RT combined with other modalities did not appear to substantially decrease QoL compared to RT alone.

## Introduction

Despite advancements in vaccination and screening programs, cervical cancer remains a significant public health issue, especially in developing countries. It is the fourth most cancer in women with 661,000 new cases and 348,000 deaths in 2022 [1]. Most patients present with early and locally advanced stages necessitating various treatment modalities [2]. Stage IA1-IA2 and selected IB1 cervical cancers can be treated with simple hysterectomy, with or without lymphatic staging, which provides a better quality of life (QoL) compared to radical hysterectomy [3]. For medically inoperable patients, radiation therapy (RT), including brachytherapy and/or external beam radiotherapy, is also an option while surgery alone is a preferred method for so-called low-risk cervical cancer due to good local control and minimal impact on QoL [4]. In cases of IB1-IB2 cervical cancer, treatment typically begins with surgery; however, about one-third of patients will require adjuvant RT [5]. On the other hand, definitive RT offers similar outcomes to surgery, with or without adjuvant radiotherapy, while combining treatments increases the risk of urological complications and lymphedema, prompting guidelines to recommend avoiding surgery if intermediate risk factors are identified at diagnosis [6,7].

Treatment strategies become even more complex for IB3-IVA cervical cancer, the most heterogeneous group. Generally, definitive chemoradiation therapy is preferred yet radical hysterectomy (type C2 or total mesometrial resection) is an option for stages IB3-IIA2 if RT is avoided due to intermediate risk factors, also known as the Sedlis criteria [7,8]. Although neoadjuvant chemotherapy (NACT) followed by surgery has not shown survival benefits over definitive chemoradiation therapy, both approaches demonstrate high cure rates, with a five-year overall survival of 72-76% [9,10]. Long-term QoL becomes a critical consideration given such survival rates and the fact that cervical cancer predominantly affects young women with an anticipated long life expectancy [11]. While treatment-related complications from surgery and RT are well-documented and often transient, the distant QoL remains a key endpoint [5,9,10]. A few studies have assessed long-term QoL between several treatment options; however, their small sample sizes, single-center settings, and limited follow-up highlight the need for further research [12-14].

## Materials and methods

Study design, data source, and setting

We conducted a cross-sectional survey-based study to evaluate the impact of various management approaches on QoL in patients with stage IA2-IIB cervical cancer. Members of the largest Russian-speaking online cervical cancer patient support group were invited to participate. Eligible participants were those over 18 years old, who had been diagnosed with stage IA2-IIB cervical cancer and had completed their treatment. The exclusion criteria were non-radical hysterectomy, pelvic exenteration, and relapse after treatment. The study was conducted from January 4, 2024, to February 28, 2024, and responses were collected via an anonymous and voluntary questionnaire, after obtaining written informed consent from each participant. Respondents were stratified into four groups based on the treatment they received: NACT followed by surgery +/- RT, surgery + RT, only RT, and only surgery.

Assessment tools

Our online survey evaluating post-management QoL was built on the official Russian versions of validated general and disease-specific European Organization for Research and Treatment of Cancer (EORTC) questionnaires - EORTC-QLQ-Core 30 (EORTC-QLQ-C30) and EORTC-QLQ-Cervical Cancer Module 24 (EORTC-QLQ-CX24) [15,16]. A high score on a functional scale indicated better functionality whereas a high score on a symptom scale/item meant greater severity of symptoms and problems. A higher score for the global health status/QoL reflected better overall QoL. Permission to use these assessment tools was obtained from the EORTC beforehand, on January 2, 2024 [17]. We supplemented them with four additional questions regarding the patient's age at the time of the survey, cancer stage, treatment received, and time elapsed since it in months.

Ethical approval

The Ethics Committee at Moscow City Oncology Hospital No. 62 determined that this study was exempt from review (Application No. 13586, dated December 25, 2023). This decision was based on the nature of our research, which involved a survey-based study with no intervention or interaction with the participants and ensured their anonymity and confidentiality. Data were collected anonymously, and informed consent was obtained from all participants prior to the survey.

Statistical analysis

Statistical analysis was performed using IBM SPSS Statistics software (Version 23.0; Armonk, NY: IBM Corporation). We interpreted reported scores using formulas from the official EORTC-QLQ-C30 and EORTC-QLQ-CX24 Scoring Manuals [18,19]. The obtained data were tested for normal distribution using the Shapiro-Wilk test and continuous variables were presented as medians with interquartile range (IQR) while categorical were reported as proportions. We analyzed the data across the groups with the Kruskal-Wallis H test for continuous variables and the Chi-square test or Fisher’s exact test for categorical data. Statistical significance was defined as p<0.05.

## Results

Overall, 173 patients were included in the study: NACT + surgery +/- RT group - 20 (11.6%), surgery + RT - 50 (28.9%), RT alone - 69 (39.9%), and surgery alone group - 34 (19.7%) (Table 1).

**Table 1:** Patient characteristics and scores as measured by the EORTC-QLQ-C30 by treatment group EORTC-QLQ-C30: the European Organization for Research and Treatment of Cancer Quality of Life Questionnaire Core 30; IQR: interquartile range; NACT: neoadjuvant chemotherapy; RT: radiation therapy; QoL: quality of life Bold p-value denotes statistical significance.

Variable	NACT + Surgery +/- RT (n = 20)	Surgery + RT (n = 50)	RT (n = 69)	Surgery (n = 34)	p-value
Age, years, median (IQR)	43 (38-48)	41 (39-46)	43 (38-46)	42 (37-47)	0.928
Time from treatment start, months, median (IQR)	28 (18-43)	25 (14-49)	20 (11-29)	14 (8-24)	<0.001
Global QoL, median (IQR)	66.7 (50.0-83.3)	66.7 (50.0-75.0)	66.7 (50.0-75.0)	83.3 (66.7-100.0)	<0.001
Functioning, median (IQR)					
Physical	70.0 (53.3-83.3)	66.7 (60.0-80.0)	66.7 (46.7-80.0)	80.0 (73.3-93.3)	<0.001
Role	66.7 (41.7-83.3)	75.0 (66.7-83.3)	66.7 (50.0-83.3)	83.3 (66.7-100.0)	0.037
Emotional	66.7 (50.0-83.3)	62.5 (41.7-75.0)	58.3 (41.7-75.0)	75.0 (50.0-83.3)	0.024
Cognitive	75.0 (50.0-83.3)	66.7 (50.0-83.3)	66.7 (50.0-83.3)	83.3 (50.0-100.0)	0.360
Social	75.0 (41.7-83.3)	66.7 (50.0-83.3)	66.7 (33.3-83.3)	83.3 (66.7-100.0)	0.006
Symptom intensity, median (IQR)					
Fatigue	44.4 (33.3-72.2)	44.4 (33.3-66.7)	55.6 (33.3-77.8)	33.3 (33.3-33.3)	0.001
Nausea and vomiting	8.3 (0.0-16.7)	16.7 (0.0-33.3)	16.7 (0.0-33.3)	0.0 (0.0-0.0)	<0.001
Pain	25.0 (0.0-50.0)	33.3 (16.7-50.0)	33.3 (16.7-50.0)	16.7 (0.0-33.3)	0.035
Dyspnea	0.0 (0.0-50.0)	0.0 (0.0-33.3)	33.3 (0.0-33.3)	0.0 (0.0-33.3)	0.017
Insomnia	33.3 (0.0-66.7)	33.3 (33.3-100.0)	66.7 (33.3-100.0)	33.3 (0.0-33.3)	<0.001
Appetite loss	0.0 (0.0-33.3)	0.0 (0.0-33.3)	33.3 (0.0-33.3)	0.0 (0.0-0.0)	<0.001
Constipation	33.3 (0.0-66.7)	33.3 (0.0-33.3)	33.3 (0.0-33.3)	33.3 (0.0-33.3)	0.742
Diarrhea	0.0 (0.0-50.0)	33.3 (33.3-66.7)	33.3 (33.3-66.7)	0.0 (0.0-0.0)	<0.001
Financial difficulties, median (IQR)	33.3 (0.0-66.7)	33.3 (0.0-66.7)	33.3 (0.0-66.7)	0.0 (0.0-33.3)	0.061

Patients in the surgery alone group had significantly better global QoL compared to the other groups (p<0.001) (Table 1). The scores of physical (p<0.001), role (p=0.037), emotional (p=0.024), and social (p=0.006) functioning were also substantially higher in the surgery alone group whereas cognitive functioning did not differ across the groups (p=0.360). The symptom intensity scale revealed the lowest severity of fatigue (p=0.001), nausea and vomiting (p<0.001), pain (p=0.035), and diarrhea (p<0.001) for the surgery-alone group despite the shortest median time from the treatment start to survey - 14 (IQR: 8-24) months (p<0.001). The RT-alone group had the most prominent symptoms of fatigue (p=0.001), dyspnea (p=0.017), insomnia (p<0.001), and appetite loss (p<0.001).

Results of the cervical cancer module of EORTC QLQ-CX24 are in Table 2. Symptom experience was the least significant in the surgery-alone group and the most considerable in the RT-alone group (p<0.001). Lymphedema was reported less frequently in the RT-alone group; however, the difference between the groups was not statistically significant (p=0.132). Notably, dissatisfaction with body image and sexual worry were significant across all groups except for the surgery alone; additionally, these patients had the least severe sexual/vaginal symptoms (p<0.001). Sexual enjoyment did not differ across the groups (p=0.988).

**Table 2:** Patient scores as measured by the EORTC-QLQ-CX24 by treatment group EORTC-QLQ-CX24: the European Organization for Research and Treatment of Cancer Quality of Life Questionnaire Cervical Cancer Module 24; IQR: interquartile range; NACT: neoadjuvant chemotherapy; RT: radiation therapy Bold p-value denotes statistical significance.

Variable	NACT + Surgery +/- RT (n = 20)	Surgery + RT (n = 50)	RT (n = 69)	Surgery (n = 34)	p-value
Time from treatment start, months, median (IQR)	28 (18-43)	25 (14-49)	20 (11-29)	14 (8-24)	<0.001
Symptom experience, median (IQR)	21.2 (12.1-28.8)	28.8 (18.2-36.4)	33.3 (21.2-48.5)	12.1 (12.1-21.2)	<0.001
Lymphedema, median (IQR)	33.3 (0.0-33.3)	33.3 (0.0-66.7)	0.0 (0.0-33.3)	33.3 (0.0-66.7)	0.132
Peripheral neuropathy, median (IQR)	66.7 (33.3-66.7)	33.3 (0.0-66.7)	33.3 (0.0-66.7)	16.7 (0.0-33.3)	0.050
Menopausal symptoms, median (IQR)	66.7 (33.3-66.7)	66.7 (33.3-100.0)	66.7 (33.3-100.0)	33.3 (0.0-66.7)	0.001
Body image, median (IQR)	50.0 (27.8-77.8)	55.6 (33.3-66.7)	55.6 (33.3-66.7)	33.3 (11.1-55.6)	0.013
Sexual worry, median (IQR)	66.7 (66.7-100.0)	66.7 (66.7-100.0)	66.7 (33.3-100.0)	33.3 (33.3-66.7)	0.018
Sexual activity, median (IQR)	33.3 (16.7-50.0)	33.3 (33.3-66.7)	33.3 (0.0-33.3)	33.3 (33.3-66.7)	<0.001
Sexual/vaginal symptoms, median (IQR)	50.0 (25.0-50.0)	33.3 (25.0-58.3)	33.3 (25.0-58.3)	16.7 (8.3-33.3)	<0.001
Sexual enjoyment, median (IQR)	33.3 (0.0-66.7)	33.3 (0.0-66.7)	33.3 (16.7-66.7)	33.3 (16.7-66.7)	0.988

Additionally, we assessed the QoL in the groups that received RT, either in combination with other modalities or alone. The median follow-up was shorter in the RT-alone group (p=0.017). Patients who received only RT reported significantly higher rates of insomnia (p=0.009). However, no differences were observed in other symptoms and functional scales of the EORTC-QLQ-C30 questionnaire between the combined treatment and RT-alone groups (Table 3). In the EORTC-QLQ-CX24 module, patients in the RT-alone group experienced lower sexual activity (p<0.001) and higher unspecified symptom burden (p=0.003) (Table 4). Overall, RT combined with other treatment modalities did not appear to substantially decrease QoL compared to RT alone.

**Table 3:** Patient characteristics and scores as measured by the EORTC-QLQ-C30 in combined treatment and RT-alone groups EORTC-QLQ-C30: the European Organization for Research and Treatment of Cancer Quality of Life Questionnaire Core 30; IQR: interquartile range; NACT: neoadjuvant chemotherapy; RT: radiation therapy; QoL: quality of life Bold p-value denotes statistical significance.

Variable	NACT + Surgery +/- RT (n = 20)	Surgery + RT (n = 50)	RT (n = 69)	p-value
Age, years, median (IQR)	43 (38-48)	41 (39-46)	43 (38-46)	0.815
Time from treatment start, months, median (IQR)	28 (18-43)	25 (14-49)	20 (11-29)	0.017
Global QoL, median (IQR)	66.7 (50.0-83.3)	66.7 (50.0-75.0)	66.7 (50.0-75.0)	0.874
Functioning, median (IQR)				
Physical	70.0 (53.3-83.3)	66.7 (60.0-80.0)	66.7 (46.7-80.0)	0.197
Role	66.7 (41.7-83.3)	75.0 (66.7-83.3)	66.7 (50.0-83.3)	0.061
Emotional	66.7 (50.0-83.3)	62.5 (41.7-75.0)	58.3 (41.7-75.0)	0.224
Cognitive	75.0 (50.0-83.3)	66.7 (50.0-83.3)	66.7 (50.0-83.3)	0.644
Social	75.0 (41.7-83.3)	66.7 (50.0-83.3)	66.7 (33.3-83.3)	0.725
Symptom intensity, median (IQR)				
Fatigue	44.4 (33.3-72.2)	44.4 (33.3-66.7)	55.6 (33.3-77.8)	0.301
Nausea and vomiting	8.3 (0.0-16.7)	16.7 (0.0-33.3)	16.7 (0.0-33.3)	0.189
Pain	25.0 (0.0-50.0)	33.3 (16.7-50.0)	33.3 (16.7-50.0)	0.535
Dyspnea	0.0 (0.0-50.0)	0.0 (0.0-33.3)	33.3 (0.0-33.3)	0.142
Insomnia	33.3 (0.0-66.7)	33.3 (33.3-100.0)	66.7 (33.3-100.0)	0.009
Appetite loss	0.0 (0.0-33.3)	0.0 (0.0-33.3)	33.3 (0.0-33.3)	0.071
Constipation	33.3 (0.0-66.7)	33.3 (0.0-33.3)	33.3 (0.0-33.3)	0.561
Diarrhea	0.0 (0.0-50.0)	33.3 (33.3-66.7)	33.3 (33.3-66.7)	0.036
Financial difficulties, median (IQR)	33.3 (0.0-66.7)	33.3 (0.0-66.7)	33.3 (0.0-66.7)	0.692

**Table 4:** Patient scores as measured by the EORTC-QLQ-CX24 in combined treatment and RT-alone groups EORTC-QLQ-CX24: the European Organization for Research and Treatment of Cancer Quality of Life Questionnaire Cervical Cancer Module 24; IQR: interquartile range; NACT: neoadjuvant chemotherapy; RT: radiation therapy Bold p-value denotes statistical significance

Variable	NACT + Surgery +/- RT (n = 20)	Surgery + RT (n = 50)	RT (n = 69)	p-value
Time from treatment start, months, median (IQR)	28 (18-43)	25 (14-49)	20 (11-29)	0.017
Symptom experience, median (IQR)	21.2 (12.1-28.8)	28.8 (18.2-36.4)	33.3 (21.2-48.5)	0.003
Lymphedema, median (IQR)	33.3 (0.0-33.3)	33.3 (0.0-66.7)	0.0 (0.0-33.3)	0.243
Peripheral neuropathy, median (IQR)	66.7 (33.3-66.7)	33.3 (0.0-66.7)	33.3 (0.0-66.7)	0.654
Menopausal symptoms, median (IQR)	66.7 (33.3-66.7)	66.7 (33.3-100.0)	66.7 (33.3-100.0)	0.268
Body image, median (IQR)	50.0 (27.8-77.8)	55.6 (33.3-66.7)	55.6 (33.3-66.7)	0.904
Sexual worry, median (IQR)	66.7 (66.7-100.0)	66.7 (66.7-100.0)	66.7 (33.3-100.0)	0.799
Sexual activity, median (IQR)	33.3 (16.7-50.0)	33.3 (33.3-66.7)	33.3 (0.0-33.3)	<0.001
Sexual/vaginal symptoms, median (IQR)	50.0 (25.0-50.0)	33.3 (25.0-58.3)	33.3 (25.0-58.3)	0.750
Sexual enjoyment, median (IQR)	33.3 (0.0-66.7)	33.3 (0.0-66.7)	33.3 (16.7-66.7)	0.968

## Discussion

Summary of main results

The primary objective of this study was to evaluate QoL in cervical cancer patients, who received various treatment modalities. Our findings indicate that patients who underwent surgical treatment alone reported the highest overall QoL and lower symptom intensity compared to those who received RT or combined treatment despite the shortest post-treatment follow-up. Physical, role, emotional, and social functioning scores were substantially higher in the surgery-alone group while cognitive functioning did not differ significantly across the groups.

Additionally, the symptom intensity scale revealed the lowest severity of fatigue, nausea and vomiting, pain, and diarrhea for the surgery-alone group. The RT-alone group exhibited the most prominent symptoms of fatigue, dyspnea, insomnia, and appetite loss. Sexual functioning was better in the surgery-alone group in almost all components, while sexual enjoyment did not differ across the groups. The rates of lymphedema seemed higher in all groups that underwent surgery but this difference was not statistically significant. Overall, the combination of treatment modalities did not substantially decrease QoL compared to RT alone.

Results in the context of published literature

Our results align with some previous studies that generally reported lower physical functioning and higher symptom scales in RT patients compared to their surgical counterparts [20-22]. Sexual functioning was also lower in groups receiving RT compared to those undergoing surgery as a part of their treatment course. Interestingly, there was a similar or lower intensity of some symptoms like fatigue, appetite loss, or diarrhea in patients treated with combined modalities compared to RT alone, which has not been widely reported before.

Other studies have evaluated the QoL of cervical cancer survivors compared to age-matched healthy cohorts. Some reports indicate that patients who had radical hysterectomy alone demonstrated overall QoL and sexual function comparable to healthy women after a few years [23]. However, another group of authors reported higher rates of pain and fatigue, lower global health status, and poorer quality of sexual life among surgical patients compared to controls without pathology [24]. These discrepancies may be due to differences in patient populations and assessment tools used. Nevertheless, these data help doctors better understand patient QoL in the long term and help patients visualize their life a few years post-treatment and anticipate QoL changes.

Strengths and weaknesses

Strengths of our work include a comprehensive evaluation of QoL across different treatment modalities, the use of validated assessment EORTC tools, and the inclusion of a diverse patient population treated in various real-world settings. These factors enhance the generalizability and relevance of our findings to clinical practice.

Our study also has a few limitations. The inclusion of active members of a virtual Russian-speaking patient society introduces potential selection bias, as older patients, less educated individuals, and those not using social media were excluded. The follow-up period was shortest for the surgery-alone group, which, combined with a modest number of patients, might be due to the relatively fast recovery after surgery and leaving the patient society, where patients typically stay when looking for information and support. With longer follow-ups in this cohort, their results might differ. This is also true for the RT-alone group when its outcomes were compared to patients with combined treatment. Additionally, there were no control groups with healthy women, so no conclusions could be drawn on patients’ post-treatment QoL compared to the healthy population.

Implications for practice and future research

The findings of our work highlight the importance of considering long-term QoL outcomes when selecting treatment modalities for cervical cancer patients. The superior QoL reported by patients undergoing surgery alone suggests that, when feasible, surgical treatment might be preferred to minimize long-term adverse effects. Current guidelines are based on data showing better local control in RT without a difference in survival [25]. However, more recent studies have demonstrated that advancements in surgical techniques lead to excellent local control for intermediate and even high-risk cases, making QoL an important endpoint to tailor cervical cancer treatment [26,27].

Future research should aim to further investigate long-term QoL outcomes in larger, more diverse populations and across different healthcare settings. Including healthy controls will also be beneficial to provide evidence on how life quality changes compared to pre-disease life. Additionally, studies should explore strategies to mitigate the negative impact of RT on QoL, such as advanced radiation techniques or supportive care interventions. By addressing these gaps, future investigations can contribute to optimizing treatment approaches and improving the overall well-being of cervical cancer survivors.

## Conclusions

This study demonstrates that patients who underwent surgical treatment alone reported the highest overall QoL and lower symptom intensity compared to those who received RT or combined treatment modalities. Physical, role, emotional, and social functioning scores were substantially higher in the surgery-alone group. The patients who received RT alone exhibited the most prominent symptoms of fatigue, dyspnea, insomnia, and appetite loss. Sexual functioning was better in the surgery-alone group in almost all aspects, with no significant difference in sexual enjoyment across the groups. When the QoL was assessed among the groups that received RT, either in combination with other modalities or alone, it did not seem to differ significantly. These findings demonstrate the impact of various treatment modalities on long-term QoL outcomes in cervical cancer survivors and highlight the importance of its consideration when choosing the optimal management strategy.
